# Autoimmune pancreatitis masquerading as a pancreatic neoplasm: A case report

**DOI:** 10.1097/MD.0000000000043359

**Published:** 2025-07-11

**Authors:** Gabrielle K. Sharbin, Max E. Edeson, Ethan Burg, Hanieh K. Hosseini, Davood K. Hosseini, Shil Patel, Jonathan D. Weinberger, Hongfa Zhu, Rosario Ligresti

**Affiliations:** a Hackensack Meridian School of Medicine, Nutley, NJ; b Thomas J. Long School of Pharmacy, Stockton, CA; c Department of Gastroenterology, Palisades Medical Center, Hackensack, NJ; d Department of Gastroenterology, Hackensack University Medical Center, Hackensack, NJ; e Department of Pathology, Hackensack University Medical Center, Hackensack, NJ.

**Keywords:** autoimmune pancreatitis, neoplasm, pancreatic mass, recurrent pancreatitis

## Abstract

**Rationale:**

Acute pancreatitis is common with potential serious sequela; representing the 5th leading cause of in-hospital mortality. Autoimmune pancreatitis (AIP) is rare, separated into type 1 and type 2 AIP. Type 1 AIP is associated with systemic immunoglobulin G4 related disease (IgG4-RD) whereas type 2 AIP is localized disease characterized by neutrophilic infiltrate. Both types of AIP can cause recurrent acute pancreatitis and chronic inflammation. On imaging, AIP can present as a focal pancreatic mass mimicking pancreatic cancer. Given the potential for misdiagnosis and unnecessary surgery, it is important for clinicians to recognize AIP in patients presenting with pancreatic masses and recurrent pancreatitis.

**Patient concerns:**

A 51-year-old female with a history of hyperlipidemia, Grave disease s/p thyroidectomy and recurrent acute pancreatitis s/p cholecystectomy presented with 3 to 4 days of epigastric pain radiating to the back with nausea, vomiting and diarrhea.

**Diagnosis:**

Labs showed elevated lipase (629 U/L) and leukocytosis. CT Abdomen and Pelvis showed pancreatitis with peri/pancreatic edema and a 1.4 cm pancreatic head hypoattenuating focus. Magnetic resonance imaging with and without contrast with magnetic resonance cholangiopancreatography (MRCP) revealed a 3.9 cm mass concerning for adenocarcinoma. Serum carbohydrate antigen 19-9 (CA-19-9) was elevated (186 U/mL), and carcinoembryonic antigen was normal. Endoscopic ultrasound showed a pancreatic head 3.4 cm heterogenous mass-like lesion. Histopathology showed neutrophilic infiltrates, microabscesses and fibrosis without malignancy consistent with AIP type 2. Serum immunoglobulin G4 was normal (20.6 mg/dL).

**Interventions:**

The patient was started on a prednisone taper of 40 mg daily.

**Outcomes:**

Her symptoms improved. Two weeks later, lipase decreased to 77 U/L and CA-19-9 was 30 U/L. Repeat MRCP showed improvement.

**Lessons:**

This case highlights the importance of AIP as a rare but critical differential for recurrent pancreatitis and pancreatic masses. Recognizing this entity is essential to increase provider familiarity with its clinical presentation when evaluating similar patients. Awareness of AIP can lead to earlier diagnosis and treatment, preventing unnecessary oncologic workups or surgery. This can reduce the risk of complications, recurrences, and lifetime cancer risk, improving patient outcomes.

## 1. Introduction

Acute pancreatitis affects ~300,000 annually in the United States, representing the 5th leading cause of in-hospital mortality.^[1–3]^ Acute pancreatitis is associated with intestinal fluid shifts, parenchymal and peri-pancreatic necrosis, and multi-organ failure.^[1–4]^ Alcohol-induced (16%–27%) and gallstones (21%–33%) represent the 2 most common etiologies.^[1–3]^ Autoimmune pancreatitis (AIP) is a rare cause of acute pancreatitis. The International consensus diagnostic criteria defines the condition as pancreatitis characterized by obstructive jaundice with or without masses, lymphoplasmacytic infiltrate/fibrosis, and marked response to steroids.^[5–9]^ AIP can be further divided into 2 types.^[5–7]^

Type 1 AIP is a manifestation of immunoglobulin G4 related disease (IgG4-RD). In addition to elevated IgG4, IgG4-RD induces infiltration of various organ systems – the pancreas commonly being affected.^[5–7]^ Serum IgG4 has poor sensitivity but high specificity, requiring biopsy showing lymphoplasmacytic infiltrate with IgG4-positive plasma cells for definitive diagnosis.^[6]^ Type 1 AIP shares many features with pancreatic cancer, both occurring in 60 to 70-year-old males, presenting as painless obstructive jaundice, with elevated CA-19-9.^[5–9]^ It can present as a focal enhancing mass in the pancreas on imaging, mimicking pancreatic cancer, but more commonly appears as a diffuse enlargement of the pancreas with delayed homogeneous enhancement.^[5–7]^

Type II AIP presents as recurrent acute pancreatitis (RAP) and more localized disease. It commonly affects middle-aged men and women.^[5–7]^ Type II AIP has a strong association with inflammatory bowel diseases (IBD) with 15% to 30% of cases being found in IBD patients.^[5,6]^ Pathology exhibits neutrophilic infiltrates without serum IgG4 association. Radiographically, type II AIP appears similar to type I. Biopsy remains the best way to differentiate type I versus type II AIP and pancreatic cancer.^[5–7]^ We present a rare case of Type II AIP mimicking a pancreatic mass on imaging.

## 2. Case description

A 51-year-old female of Latina descent with hypertension, hyperlipidemia, Grave disease, and RAP, presented with 3 to 4 days of abdominal pain, suspicious for acute on chronic pancreatitis. She endorsed epigastric pain radiating to the back, nausea, vomiting, and diarrhea; and denied fever, chills, fatigue, dyspnea, dizziness, bloody stool/melena, recent weight loss, or other gastrointestinal symptoms. No new medications, recent infections, trauma, or family history of similar illness were reported. She has 1 to 2 drinks per month. She has had ~5 other episodes of pancreatitis without diagnosis. Given her recurrent episodes of pancreatitis a cholecystectomy was previously performed for possible gallstones causing her pancreatitis, however this did not result in cessation of her symptoms. Fifteen years prior, during her first episode of pancreatitis, she had been worked up for AIP with negative serum IgG4. Endoscopic ultrasound (EUS) with fine needle biopsy (FNB) at the time showed an enlarged pancreatic body, concerning for AIP, however, pathology results were negative. Although, a lack of intact pancreatic ducts or islets in the provided samples rendered this evaluation limited and therefore not entirely excluding a diagnosis of AIP. On presentation, the patient was tachycardic, hypertensive and afebrile, uncomfortable appearing but not in distress with epigastric tenderness but no rebound, guarding, or signs of peritonitis. Labs showed leukocytosis, elevated lipase (629 U/L), normal liver and metabolic panels, and elevated CA-19-9 levels (186 U/mL) with normal serum carcinoembryonic antigen (CEA).

**Figure 1.**
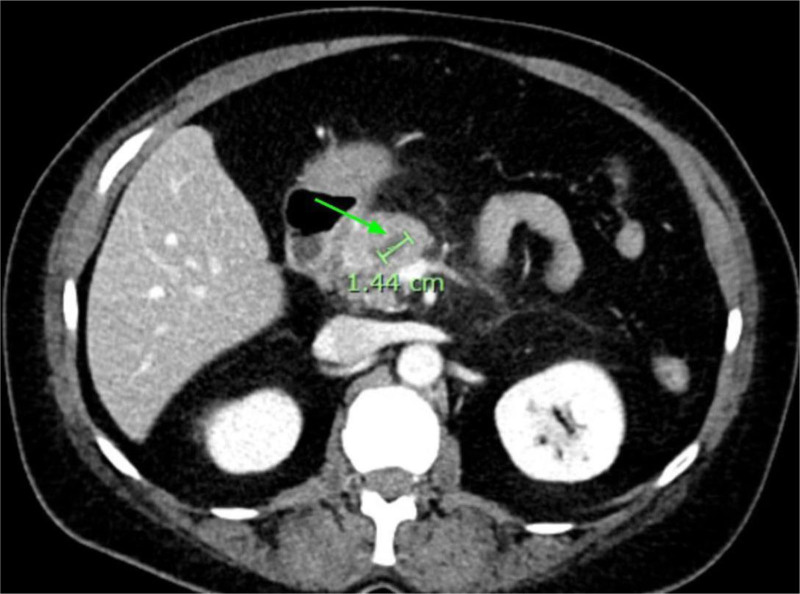
Computed tomography of abdomen and pelvis with contrast. CT revealing a 1.4 cm hypoattenuating focus in the pancreatic neck (green arrow) consistent with either a mass, developing necrosis or focal edematous change.

**Figure 2.**
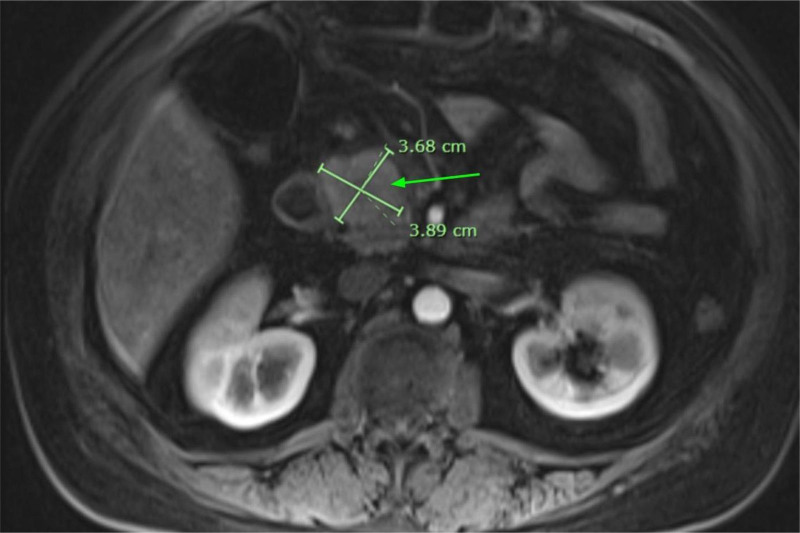
Magnetic resonance imaging abdomen with and without contrast with magnetic resonance cholangiopancreatography. MRI of the abdomen revealed a 3.9 cm mass on the head of the pancreas (green arrow) concerning for adenocarcinoma versus focal inflammatory change. MRI = magnetic resonance imaging.

**Figure 3.**
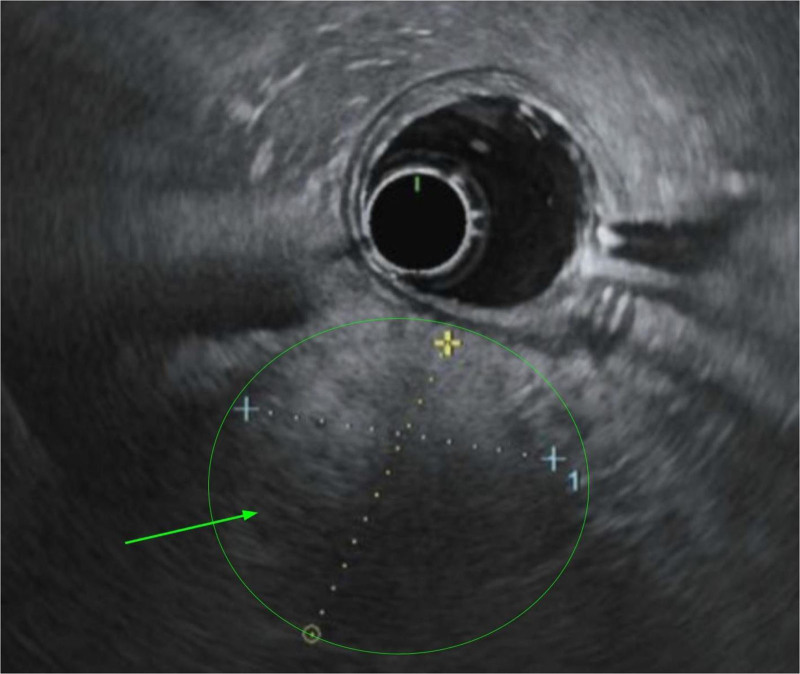
Endoscopic ultrasound of the pancreatic mass. EUS showed 3.4 cm heterogenous mass-like area in the pancreatic head (green arrow) concerning autoimmune changes versus malignancy. The mass-like area was subsequently biopsied. EUS = endoscopic ultrasound.

**Figure 4.**
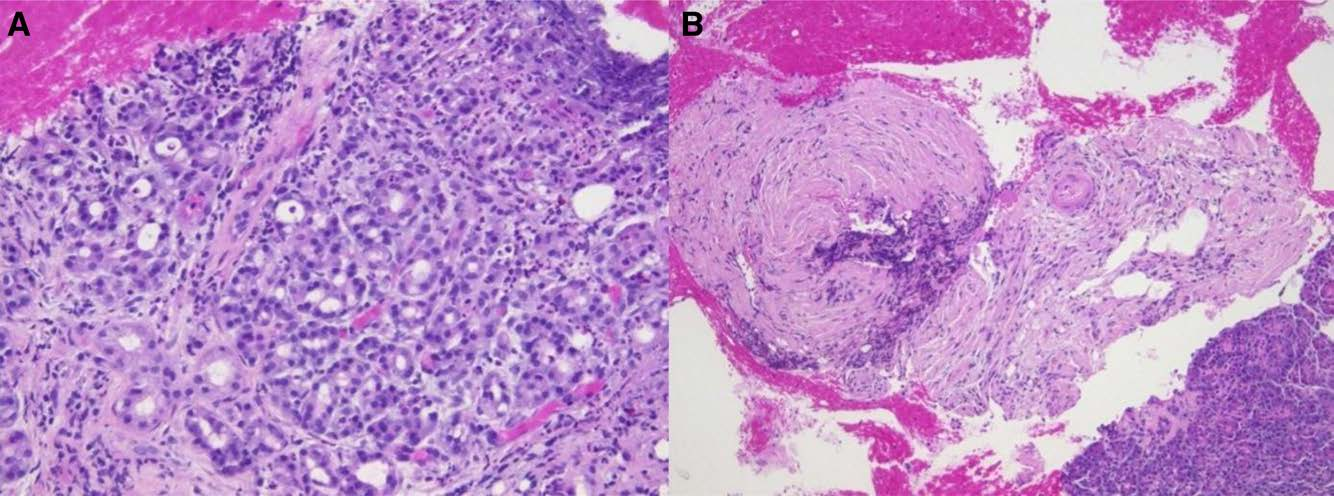
Pancreatic endoscopy ultrasound biopsy pathology. (A) Hematoxilin and eosin (H&E) stained pancreatic parenchyma at 200× magnification with predominantly neutrophilic infiltrate consistent with autoimmune pancreatitis type 2. (B) H&E stained pancreatic stroma at 100× magnification showing stromal fibrosis and mixed inflammatory infiltrate with eosinophils.

CT Abdomen and Pelvis showed hepatic steatosis and pancreatitis with peri/pancreatic edema (Fig. 1). CT also showed a 1.4 cm pancreatic head hypoattenuating focus requiring further imaging to differentiate between a mass, necrosis, or focal edematous change (Fig. 1). Magnetic resonance imaging (MRI) with and without contrast with magnetic resonance cholangiopancreatography (MRCP) showed a 3.9 cm mass concerning for adenocarcinoma (Fig. 2). The patient underwent EUS with FNB using a Limaca 20 gauge motorized needle, with 2 samples being taken for diagnosis. EUS showed a pancreatic head 3.4 cm heterogenous mass-like lesion, peri-pancreatic fluid collection, and abdominal ascites (Fig. 3). Histopathology showed neutrophilic infiltrates, microabscesses and fibrosis without evidence of malignancy consistent with AIP type 2 (Fig. 4). Serum immunoglobulins were evaluated to further characterize AIP, with all levels in normal range, including IgG4 (20.6 mg/dL). The patient began a 40 mg oral prednisone taper with rapid symptomatic relief, further confirming the diagnosis of autoimmune pancreatitis. After 2 weeks of steroid therapy, repeat laboratory studies showed significant improvement with lipase decreasing from 629 U/L to 77 U/L and CA-19-9 decreasing from 186 U/mL to 30 U/mL. Liver enzymes and CEA remained within normal limits. She tolerated the taper well on outpatient follow-up with resolution of symptoms. Repeat MRCP performed 6 weeks post discharge revealed an interval decrease in size and volume of the pancreas, with resolution of peri-pancreatic edema. The imaging findings were consistent with a resolving episode of acute autoimmune pancreatitis. It was recommended to repeat the MRI in 3 to 6 months.

## 3. Discussion

AIP should be considered in recurrent pancreatitis despite its rarity and diagnostic pitfalls.^[6]^ Further data is essential to better characterize AIP, and help differentiate it from malignancy. AIP often has histological patterns which are diagnostic and pivotal. The diagnosis requires any combination of one or more features of AIP.^[9]^ These include: imaging of pancreatic parenchyma and pancreatic ducts, serology, other organ involvement, histopathology of the pancreas, and response to steroids.^[9]^ Our case highlights the limitations of imaging as MRI and CT showed a focal-like mass which was found to be an inflammatory collection.

Diffuse enlargement of the pancreas with a capsule-like rim and irregular narrowing of the main pancreatic duct are characteristic findings in AIP.^[10]^ However, AIP can often appear as a mass-like focal lesion.^[11]^ Biopsy and multiple rounds of imaging may be necessary to differentiate malignancy from focal inflammation due to similarity in presentation. Patient specific risk factors should be assessed when considering malignancy versus AIP. Risk factors to evaluate for pancreatic cancer include smoking, obesity, diabetes, alcohol intake, and chronic pancreatitis,^[12]^ while AIP risks include autoimmune disease (IBD for type II AIP), family history, and history of recurrent pancreatitis.^[6]^

RAP as seen in our patient can be difficult to manage, particularly in patients where no apparent cause is identified. An organizing committee was formed to devise recommendations regarding the diagnosis and management of patients with idiopathic acute recurrent pancreatitis (IARP) and attempt to establish a standard of care.^[13]^ It should be noted that the standard of care includes a multitude of serological and imaging studies which may not be feasible to obtain in most medical settings. Additionally, the standard may not be sufficient to correctly diagnose all patients as the etiology of RAP remains unclear in 10% to 15% of patients despite a thorough evaluation.^[13]^ First it is important to correctly identify patients with IARP. The diagnosis of acute pancreatitis is made based on 2 of the the 3 following criteria: clinical (upper abdominal pain), laboratory (serum amylase or lipase ≥3× the upper limit of normal), imaging suggestive of inflammation (CT, MRI, ultrasound).^[13]^ IARP is defined as RAP after apparent causes have been excluded via history, routine lab tests (i.e., triglycerides, calcium), imaging (EUS, MRCP, ERCP with or without manometry), or genetic testing.^[13,14]^

Once it has been determined that a patient has RAP, the underlying etiology should be investigated. Investigation of an underlying etiology after common causes such as alcohol and gallstones have been ruled out can be difficult and costly. In these cases the Toxic-metabolic, Idiopathic, Genetic, Autoimmune, Recurrent and severe acute pancreatitis and Obstructive (TIGAR-O) Pancreatitis Risk/Etiology Checklist can serve as a useful framework in investigating an underlying cause.^[14]^ It should also be noted that more than 1 potential etiology may be present in the same patient. Toxic-metabolic causes such as hypertriglyceridemia and hypercalcemia can be easily tested for with serological studies.^[14]^ There should be a high index of suspicion for medication induced RAP, despite its rarity and despite the difficulty in identifying potentially offending medications (particularly in patients on multiple medications).^[15]^

While pancreas divisum is not a clearly established etiology of RAP, genetic mutations and polymorphisms may play a role in causing pancreatitis in patients with pancreas divisum.^[13,14]^ Therefore, genetic mutations should be investigated in these patients. Commonly identified mutations in the CFTR, SPINK1 and PRISS1, CTRC genes have been associated with pancreatitis and should be evaluated in patients younger than 35 with RAP.^[13,14]^ In particular mutations in PRISS1 is a well characterized autosomal dominant cause of hereditary RAP.^[14]^ Finally, evaluation of RAP should include assessment of unusual etiologies such as ampullar neoplasms and anatomic variants like type III choledochal cyst, and anomalous pancreaticobiliary junction.^[13,14]^

EUS is the most sensitive test for investigating occult gallstones or microlithiasis, and small or occult tumors associated with RAP. Therefore, following standard imaging (CT, ultrasound), EUS should be the first line test in evaluating patients with RAP.^[13,14]^ Contrast enhanced CT is the first line cross-sectional imaging test for evaluating RAP.^[13,14]^ MRI/MRCP is the most accurate cross-sectional imaging test in detecting pancreatic ductal abnormalities that may contribute to or result from RAP and secretin stimulation significantly improves the diagnostic yield of intraductal abnormalities that might contribute to or result from RAP.^[13,14]^ It is recommended that patients with acute recurrent episodes of idiopathic pancreatitis undergo a repeat abdominal ultrasound as the inflammatory milieu of pancreatitis can often lead to cholelithiasis being missed in the acute inpatient setting.^[14]^ Current guidelines recommend cholecystectomy in surgical candidates following a second episode of acute pancreatitis with no apparent cause as it drastically lowers their rate of subsequent episodes.^[1]^ However some studies recommended against empiric cholecystectomy in patients with normal LFTs and no evidence of gallbladder disease by imaging.^[13,14]^

Despite our patient’s cholecystectomy she continued to have recurrent episodes of acute pancreatitis. In patients such as this, with an unclear etiology of pancreatitis, the TIGAR-O framework suggests that rarer causes such as AIP should be investigated. In patients at higher risk, such as those with IBD or other autoimmune conditions (as seen in this case), evaluation for AIP should be prioritized before exploring the other aforementioned rare causes.^[13,14]^ Type II AIP is much more likely to present as RAP than type I (IgG4 related) AIP. Routine serological testing for IgG4 should not be performed in patients with RAP without imaging suggestive of AIP and empiric trials of steroids should not be performed for IARP without compelling evidence for AIP.

Additionally, EUS guided biopsy can provide crucial tissue data in diagnosing AIP and differentiating it from malignancy, but is not without its pitfalls as exemplified by this patient.^[16]^ Biopsy remains a definitive modality in differentiating AIP from pancreatic cancer, particularly in type 2 AIP where the serological markers and multi-organ involvement found in type 1 AIP is absent. The International consensus diagnostic criteria only considered tissue specimens from core biopsies or surgical resection as sufficient for histological diagnosis of AIP.^[17]^ Modalities such as fine needle aspiration (FNA) have a high sensitivity for detecting pancreatic cancer, but lack sufficient preservation of tissue architecture key to the diagnosis of AIP.^[17]^ Conversely FNB allows for preservation of tissue architecture and has a reported 60.20% sensitivity for AIP.^[18]^ As such, FNA may be better served to rule out pancreatic cancer in AIP patients.^[19]^ However FNB still lacks a high sensitivity for AIP and a negative biopsy should not rule out the diagnosis especially in a patient with a high index of suspicion for the disease.^[18,19]^ As demonstrated by our patient’s previous FNB being negative for AIP, this modality can miss AIP if sufficient pancreatic tissue samples are not obtained.^[17]^ While histology is not required to diagnose type I AIP, tissue confirmation is generally required in type II.^[20]^ Patients with recurrent pancreatitis without clear etiology should undergo EUS evaluation.^[21]^ Despite the initial negative biopsy, repeat biopsy proved to be essential in our case. This emphasizes both the pitfalls and utility of early biopsy to prevent recurrent pancreatitis and decrease the risk of pancreatic cancer. Still, the decision to perform biopsy remains complex given that the procedure can worsen pancreatitis.^[22]^

Recurrent episodes of acute pancreatitis can develop into chronic pancreatitis. Chronic pancreatitis is a risk factor for pancreatic cancer.^[23]^ However, the association between AIP and pancreatic cancer is controversial.^[23]^ There are a few studies investigating the relation between AIP and pancreatic cancer, but these state that association cannot be excluded.^[23]^ Th2 immunological dominance, type II macrophage polarization and basophil infiltration in type I AIP, and immunosuppressive therapies can promote the development of pancreatic cancer.^[24]^ Therefore, patients with type I AIP might benefit more from cancer surveillance. Age > 60 and serum CA-19-9 levels above 100 U/mL are considered independent risk factors for pancreatic cancer,^[24]^ and additional screening should be recommended in these cases.

## 4. Limitations

This case report has limitations inherent to its study design. Given that the report follows 1 patient, its findings may not be generalizable to broader populations. While the patient ultimately responded well to corticosteroids, definitive diagnosis relied on biopsy results, which can vary based on sampling adequacy and pathologist interpretation. Additionally, the patient’s earlier negative FNB highlights the potential for sampling error. Finally, the retrospective nature of the clinical interpretation may introduce bias, and long-term follow-up will be needed to fully assess recurrence or progression to chronic pancreatitis or malignancy.

## 5. Conclusion

This case highlights the diagnostic challenges of type II AIP, particularly in patients with recurrent pancreatitis, mass-like consolidations on imaging, and a history of autoimmune disease. Despite laboratory results being unrevealing and imaging suggesting pancreatic cancer, the patient was ultimately diagnosed with type II AIP, emphasizing the limitations of radiologic and serologic evaluation alone. Tissue sampling proved crucial in establishing the correct diagnosis, avoiding unnecessary oncologic intervention, and guiding effective corticosteroid therapy. The patient had a favorable clinical outcome with resolution of symptoms and normalization of imaging and laboratory markers following steroid treatment. However, repeated episodes of acute pancreatitis carry a known risk of progression to chronic pancreatitis, and pancreatic malignancy. This case underscores the importance of tissue diagnosis and the utility and limitations of FNA and FNB in diagnosis of AIP. Biopsy proved to be crucial in this patient due to the uncertainty of serology and imaging and the need to differentiate AIP from pancreatic cancer. This is crucial for rapid diagnosis and treatment, preventing unnecessary work-up, and reducing complications and cancer development from recurrent pancreatitis. Moreover, this case provides a diagnostic framework for IARP and highlights its feasibility within a healthcare system. This report contributes to the limited literature on type II AIP, supporting the need for further research to improve diagnostic criteria and management strategies for this rare disease.

## Author contributions

**Conceptualization:** Davood K. Hosseini, Max E. Edeson, Ethan Burg, Hanieh K. Hosseini, Jonathan D. Weinberger, Rosario Ligresti.

**Data curation:** Davood K. Hosseini, Gabrielle K. Sharbin, Hongfa Zhu.

**Formal analysis:** Davood K. Hosseini.

**Investigation:** Davood K. Hosseini, Ethan Burg, Hanieh K. Hosseini, Jonathan D. Weinberger, Rosario Ligresti.

**Methodology:** Davood K. Hosseini.

**Software:** Davood K. Hosseini.

**Supervision:** Davood K. Hosseini, Rosario Ligresti.

**Validation:** Davood K. Hosseini.

**Writing – original draft:** Davood K. Hosseini, Gabrielle K. Sharbin, Max E. Edeson, Ethan Burg, Hanieh K. Hosseini, Shil Patel, Jonathan D. Weinberger, Hongfa Zhu, Rosario Ligresti.

## References

[1] Tenner S Vege SS Sheth SG. American College of Gastroenterology guidelines: management of acute pancreatitis. Am J Gastroenterol. 2024;119:419–37. 38857482 10.14309/ajg.0000000000002645 PMC13221274

[2] Mederos MA Reber HA Girgis MD. Acute pancreatitis: a review. JAMA. 2021;325:382–90. 33496779 10.1001/jama.2020.20317

[3] Lankisch PG Apte M Banks PA. Acute pancreatitis. Lancet. 2015;386:85–96. 25616312 10.1016/S0140-6736(14)60649-8

[4] Fu CY Yeh CN Hsu JT Jan YY Hwang TL. Timing of mortality in severe acute pancreatitis: experience from 643 patients. World J Gastroenterol. 2007;13:1966–9. 17461498 10.3748/wjg.v13.i13.1966 PMC4146974

[5] Goyal S Sakhuja P. Autoimmune pancreatitis: current perspectives. Indian J Pathol Microbiol. 2021;64(Supplement):S149–59. 34135159 10.4103/ijpm.ijpm_59_21

[6] Nista EC De Lucia SS Manilla V. Autoimmune pancreatitis: from pathogenesis to treatment. Int J Mol Sci. 2022;23:12667. 36293522 10.3390/ijms232012667 PMC9604056

[7] Masood M. Autoimmune pancreatitis: what we know so far. JGH Open. 2021;6:3–10. 35071782 10.1002/jgh3.12688 PMC8762623

[8] Gupta RK Sakhuja P Govind H Agarwal AK. Does IgG4 level evaluation in pancreatic mass play role in avoiding major surgery in uncertain presentation: a case report. Indian J Pathol Microbiol. 2020;63:282–5. 32317534 10.4103/IJPM.IJPM_289_19

[9] Shimosegawa T Chari ST Frulloni L; International Association of Pancreatology. International consensus diagnostic criteria for autoimmune pancreatitis: guidelines of the International Association of Pancreatology. Pancreas. 2011;40:352–8. 21412117 10.1097/MPA.0b013e3182142fd2

[10] Takahashi M Fujinaga Y Notohara K; Working Group Members of The Research Program on Intractable Diseases from the Ministry of Labor, Welfare of Japan. Diagnostic imaging guide for autoimmune pancreatitis. Jpn J Radiol. 2020;38:591–612. 32297064 10.1007/s11604-020-00971-z

[11] Wakabayashi T Kawaura Y Satomura Y. Clinical and imaging features of autoimmune pancreatitis with focal pancreatic swelling or mass formation: comparison with so-called tumor-forming pancreatitis and pancreatic carcinoma. Am J Gastroenterol. 2003;98:2679–87. 14687817 10.1111/j.1572-0241.2003.08727.x

[12] Klein AP. Pancreatic cancer epidemiology: understanding the role of lifestyle and inherited risk factors. Nat Rev Gastroenterol Hepatol. 2021;18:493–502. 34002083 10.1038/s41575-021-00457-x PMC9265847

[13] Guda NM Muddana V Whitcomb DC. Recurrent acute pancreatitis: international state-of-the-science conference with recommendations. Pancreas. 2018;47:653–66. 29894415 10.1097/MPA.0000000000001053

[14] Guda NM Trikudanathan G Freeman ML. Idiopathic recurrent acute pancreatitis. Lancet Gastroenterol Hepatol. 2018;3:720–8. 30215363 10.1016/S2468-1253(18)30211-5

[15] Weissman S Aziz M Perumpail RB Mehta TI Patel R Tabibian JH. Ever-increasing diversity of drug-induced pancreatitis. World J Gastroenterol. 2020;26:2902–15. 32587438 10.3748/wjg.v26.i22.2902 PMC7304112

[16] Notohara K Kamisawa T Fukushima N. Guidance for diagnosing autoimmune pancreatitis with biopsy tissues. Pathol Int. 2020;70:699–711. 32767550 10.1111/pin.12994

[17] de Pretis N Crinò SF Frulloni L. The role of EUS-guided FNA and FNB in autoimmune pancreatitis. Diagnostics (Basel). 2021;11:1653. 34573995 10.3390/diagnostics11091653 PMC8470670

[18] Chhoda A Rustagi T. EUS-guided needle biopsy for autoimmune pancreatitis. Clin J Gastroenterol. 2020;13:669–77. 32519311 10.1007/s12328-020-01153-0

[19] Sugimoto M Takagi T Suzuki R. Endoscopic ultrasonography-guided fine needle aspiration can be used to rule out malignancy in autoimmune pancreatitis patients. J Ultrasound Med. 2017;36:2237–44. 28670760 10.1002/jum.14265

[20] Zen Y. Type 2 autoimmune pancreatitis: consensus and controversies. Gut Liver. 2022;16:357–65. 34670874 10.5009/gnl210241 PMC9099380

[21] Strand DS Law RJ Yang D Elmunzer BJ. AGA clinical practice update on the endoscopic approach to recurrent acute and chronic pancreatitis: expert review. Gastroenterology. 2022;163:1107–14. 36008176 10.1053/j.gastro.2022.07.079

[22] Siddiqui AA Shahid H Shah A. High risk of acute pancreatitis after endoscopic ultrasound-guided fine needle aspiration of side branch intraductal papillary mucinous neoplasms. Endosc Ultrasound. 2015;4:109–14. 26020044 10.4103/2303-9027.156728 PMC4445167

[23] Poddighe D. Autoimmune pancreatitis and pancreatic cancer: epidemiological aspects and immunological considerations. World J Gastroenterol. 2021;27:3825–36. 34321847 10.3748/wjg.v27.i25.3825 PMC8291014

[24] Kim HS Gweon TG Park SH Kim TH Kim CW Chang JH. Incidence and risk of pancreatic cancer in patients with chronic pancreatitis: defining the optimal subgroup for surveillance. Sci Rep. 2023;13:106. 36596818 10.1038/s41598-022-26411-8 PMC9810784

